# Flame Retardants at the Top of a Simulated Baltic Marine Food Web—A Case Study Concerning African Penguins from the Gdansk Zoo

**DOI:** 10.1007/s00244-014-0081-z

**Published:** 2014-09-16

**Authors:** Andrzej R. Reindl, Lucyna Falkowska

**Affiliations:** Department of Marine Chemistry and Environmental Protection, Faculty of Oceanography and Geography, University of Gdansk, Al. Pilsudskiego 46, 81-378 Gdynia, Poland

## Abstract

The present study estimated hexabromocyclododecane (HBCD) as a sum of three main isomers (*α*, *β*, and *γ*) and tetrabromobisphenol A (TBBPA) in African penguins (*Spheniscus demersus*) from Gdansk Zoo and in their sole food, Baltic herring (*Clupea harengus*), from Gdansk Bay. The average concentration of HBCD in whole herring was 22.0 ± 9.9 ng/g lw, whereas TBBPA was approximately 10-fold lower (2.3 ± 1.3 ng/g lw). Brominated flame retardants (BFRs) were also found in muscle and liver of herring. The estimated daily dietary exposure of the penguins to HBCD was 252.9 ± 113.7 ng, whereas for TBBPA it was 26.3 ± 14.9 ng. The ability of BFRs to accumulate in the liver, muscles, fatty tissue, and brain of penguin was confirmed. The highest concentrations of HBCD (326.9 ng·g^−1^ lw) and TBBPA (14.8 ng·g^−1^ lw) were found in the brain of an adult penguin. The strongest accumulation factor for BFRs was also established for brain tissue, but it showed stronger magnification in muscle than in liver. HBCD and TBBPA were found in penguin guano and eggs, thus showing effective removal from the birds’ systems. BFRs content in yolk was approximately ten times greater than in albumen indicating the lipophilic character of these compounds.

Brominated flame retardants (BFRs) are persistent, bioaccumulative and toxic chemicals that can travel a long distance and have been widely used for approximately 30 years (Law et al. 2006; de Wit et al. 2010). These substances are present in aquatic ecosystems where they accumulate in fish and may subsequently biomagnify in marine food webs (de Wit 2002; Guerra et al. 2009; Shaw et al. 2009; Bignert et al. 2011).

Polybrominated diphenyl ethers are one of the common groups of flame retardants. The most popular of these is tetrabomobisphenol A (TBBPA), which accounts for approximately 75 % of the Asian market and approximately 59 % of the global market in terms of use. In Europe, the most common is hexabromocyclododecane (HBCD), which constitutes approximately 57 % of European consumption and approximately 8 % of all flame retardants used in the world. Although most major manufacturers of BFRs are based in the United States, HBCD is also produced in Europe (Holland) (de Wit 2002; Law et al. 2006; HELCOM 2010).

TBBPA is used both as a reactive form and as an additive in other polymer applications. In its reactive form, which is often used in printed circuit boards, it draws a covalent reaction from the phenolic hydroxyl group and is incorporated into the polymer matrix. The additive form, in which it remains independent of the polymer structure, TBBPA is used in akrylonitrile butadiene styrene, a common constituent of electronic equipment (Morose 2006). It is estimated that approximately 10 % of TBBPA used worldwide is as an additive retardant. In addition, TBBPA is used in the synthesis of other retardants such as brominated epoxy oligomers. HBCD is an additive form of flame retardant that is added to expanded polystyrene (United States Environmental Protection Agency) and extruded polystyrene as well as high-impact polystyrene). It is also used in textiles to decrease flammability. The technical product is a mixture of three main isomers, among which 70–90 % are constituted of *γ*-HBCD and the remaining 10–30 % of *α*- and *β*-HBCD isomers (de Wit 2002; Alaee et al. 2003; HELCOM 2009). In biological material, the predominant isomer is *α*-HBCD, which is characterized by greater durability in the environment (Covaci et al. 2006; Kuiper et al. 2007; Janák et al. 2008).

The chemical and physical properties of HBCD and TBBPA show similarities with currently known persistent organic pollutants (POPs), and both of these compounds are potentially classified as persistent, bioaccumulative, and toxic). Moreover, their lipophilic nature (log Kow_(technical HBCD)_ = 5.6 or and log Kow_(TBBPA)_ = 5.9) means that they accumulate in the fat of organisms including fish and piscivorous animals (Remberger et al. 2004). HBCD may also induce cancer by way of a nonmutagenic mechanism and cause skin sensitisation and inhibition of plasma membrane uptake of the neurotransmitters dopamine, glutamate, and *γ*-amino-*n*-butyric-acid. Furthermore, it may act as a peroxisome proliferator, which is a mechanism implicated in nonmutagenic cancer (Sellström et al. 2003), and it is suspected of affecting thyroid hormone mediate gene expression and of being a potential endocrine disruptor (Janák et al. 2008; Kakimoto et al. 2008; de Wit et al. 2010).

Durability in the environment, resistance to degradation, and ability to accumulate in food webs all make BFRs potentially dangerous to humans. The presence of some flame retardants has been determined in the milk of breastfeeding mothers (Covaci et al. 2006; Kakimoto et al. 2008). The most significant route of exposure to POPs is through diet. The aim of the present study was to analyze TBBPA and HBCD (*α*, *β*, *γ* isomers) in African penguins (*Spheniscus demersus*) from the Gdansk Zoo and their primary food source, i.e., Baltic herring (*Clupea harengus*) from Gdansk Bay. The following aspects were analyzed: (1) the penguins’ ability to accumulate HBCD and TBBPA in different tissue and organs and (2) the penguins’ ability to excrete HBCD and TBBPA.

## Materials and Methods

### Sample Collection

Herring (*C. harengus*) from the coastal zone of the Southern Baltic constitute the sole food of the African penguins (*S. demersus*) living in Gdansk Zoo. Such fish (*n* = 9), weighing between 46.90 g and 192.13 g (wet mass), were selected for testing. Additional fish were collected and prepared to obtain skinless muscle tissue (*n* = 6) and livers (*n* = 6). The collection of fish was performed once a month from December 2009 to February 2010. Guano was collected from the Teflon mat covering the enclosure at the zoo. One sample for the analysis of BFRs was composed of the guano of several specimens. Unhatched penguin eggs displaying no symptoms of decomposition were obtained during the breeding seasons of autumn 2009 and spring 2010, placed in polyethylene bags, and transported to the laboratory where tests were performed on whole eggs (*n* = 3), yolks (*n* = 3), and albumen (*n* = 3). Samples of breast muscle, liver, fatty tissue, and brain were obtained from dead birds (*n* = 3). All samples were homogenized, lyophilized, and then homogenized once again in an etched porcelain mortar before being placed in glass jars.

### Reagents and Standards

Standards for TBBPA and the *α*-, *β*- and *γ*-HBCD isomers used in the procedure were supplied by AccuStandard (purity high-performance liquid chromatography [HPLC] area ≥99.3–100 %). The extraction solvents used during chromatographic analysis, *n*-hexane (purity gas chromatography [GC] area ≥99.0 %) and acetone (purity GC area ≥99.9 %), were supplied by Merck. The following were also used as part of the procedure: sulphuric acid (minimum 95 %) and nitric acid (65 %) (both ultra-analytically pure) from Poch, acetonitrile hypergrade for LC-mass spectrometry LiChrosolv and high-purity water from MERCK, SPE columns with magnesium silicate (LC-Florisil) from Thermo Scientific HyperSep, rounded cellulose extraction thimbles from Whatman, and 99.994 % pure nitrogen from Linde.

### Quality Control and Assurance

When performing analyses, vessels and containers that came into contact with the analyzed organic samples were thoroughly washed and etched in 65 % HNO_3_. The BFRs were recovered by adding the known standard concentrations for TBBPA and mix of the *α*-, *β*- and *γ*-HBCD isomers to the biological sample material before extraction. The analytical method used made it possible to recover 91.0–93.8 % of TBBPA and 86.3–90.1 % of the HBCD isomers. In the analytical procedure, tests were performed on blank samples to prove that HBCD and TBBPA had not been introduced at the stage of sample preparation and purification. The quantitation limit for the studied compounds was 0.9 and 1.4 µg·kg^−3^ for TBBPA and HBCD, respectively.

### Sample Preparation and Extraction

Extraction was performed using Soxhlet method with a 1:1 *n*-hexane:acetone mixture. The extract was purified in a reaction with concentrated H_2_SO_4_ to remove lipids, and final purification was performed using SPE method on type C18 cartridges (Remberger et al. 2004). Elution was performed with the extraction solvent.

### Chemical Analyses

TBBPA and HBCD determination was performed using HPLC in accordance with methodology described elsewhere (Vilaplana et al. 2009; Köppen et al. 2008) on the Agilent 1200 apparatus series, equipped with diode array detector, and run at a fixed wavelength of 208 nm. Separation was performed on a combined chromatography column: Zorbax Eclipse XDB C18 by Agilent and a chiral Nucleodex *β*-PM. For the mobile phase of chromatographic analysis, a solution of acetonitrile and high purity water (8:2) with a flow rate of 1 ml·min^−1^ was used.

### Statistical Modelling

Results were calculated as average concentrations with SD, and correlation coefficients were determined using Spearman’s test.

## Results and Discussion

**Fig. 1:**
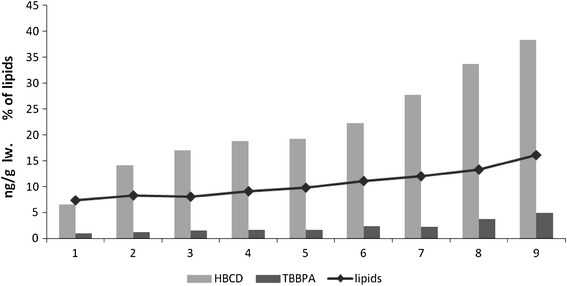
HBCD concentrations and lipid content in whole herring (*C. herengus*) caught in the coastal zone of the southern Baltic (2009–2010)

**Fig. 2:**
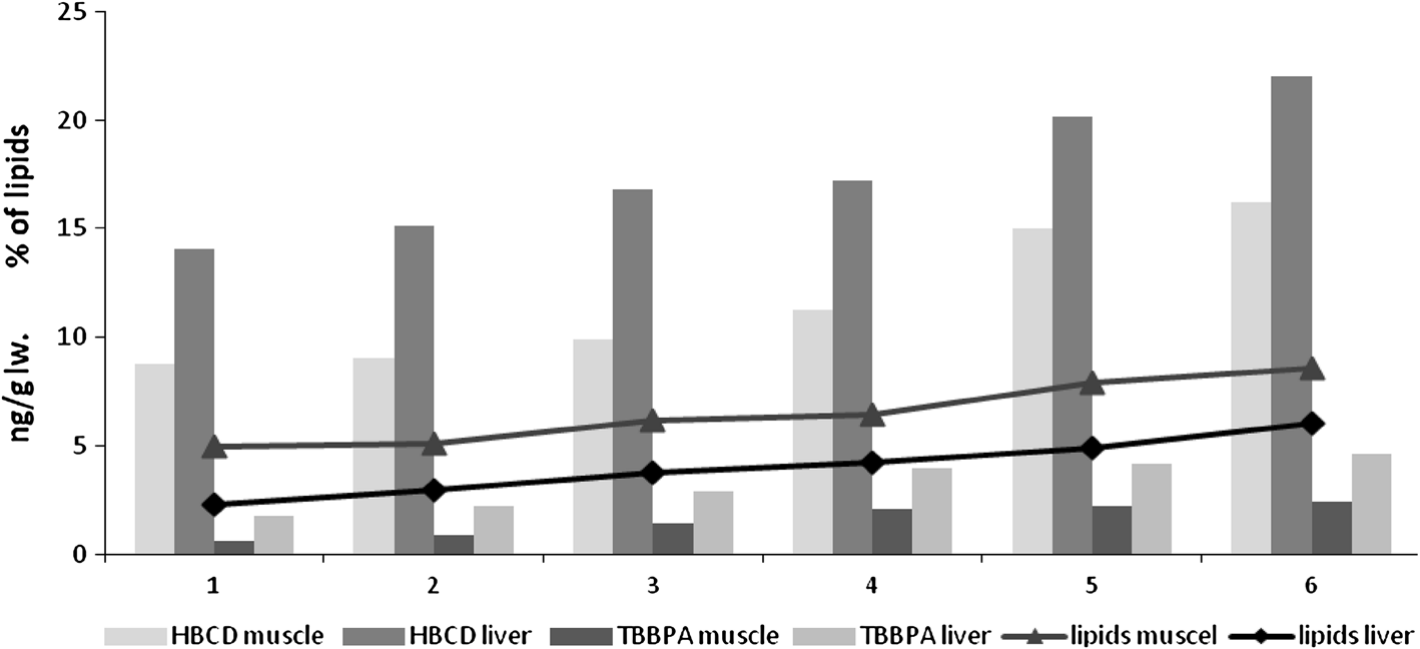
HBCD concentrations and lipid content in the muscles and livers of herring caught in the coastal zone of the southern Baltic (2009–2010)

The Baltic herring is a fatty fish, the fat tissue growth of which accompanies growth in body mass. The lowest lipid content (7.4 %) was determined in the smallest fish, which weighed 46.9 g, and it was possible to observe a directly proportional increase ≤16.1 % in fish of the highest mass, i.e., 192.1 g. In this regard, lipid content increased (*r* = 0.97, *p* < 0.05) with increasing body weight. The analyzed BFRs, both of which were found to be present in Southern Baltic herring, followed a similar pattern. The concentrations of these compounds were noted to increase with the size of the fish and also with lipid content (*r*_HBCD_ = 0.96; *r*_TBBPA_ = 0.97; *p* < 0.05; Fig. 1). Concentrations of both TBBPA and HBCD were also observed to increase together with lipid content in localized samples of herring muscle and liver (Fig. 2). The lipophilic properties of these BFRs and the fact that brominated compounds can accumulate in the tissues and internal organs of fish suggests their similarity to other POPs (Remberger et al. 2004).

The results of a study by Bignert et al. (2011) indicate temporal and spatial changeability of HBCD concentrations in muscle of herring caught in various regions of the Baltic. However, whereas HBCD concentrations in herring muscle decrease, in blue mussels the decreasing trend is nonsignificant. Tests performed by Janák et al. (2008) indicated concentrations of HBCD at a level of 26 ng·g^−1^ lw. A similar concentration of HBCD was found in muscle of herring from the southern Baltic Proper ranging between 21 and 38 ng·g^−1^ lw with an average concentration of 30 ng·g^−1^ lw (HELCOM 2009).

Research performed by Remberger et al. (2004) indicated significantly greater HBCD levels downstream from the waste outlet of a Swedish textile factory than upstream with levels for pike and eel varying between 65 and 1808 ng·g^−1^ lw. It was also noted that muscle of herring caught between 1999 and 2000 in an HBCD-contaminated area of the Northern Baltic contained between 21 and 180 ng·g^−1^ lw of this compound, whereas the average value for muscle of herring caught in 2001 in the “pure” zone of the Gulf of Bothnia was somewhere between 10 and 20 ng·g^−1^ lw. HBCD content determined in muscle of herring caught in 2009–2010 in the Polish waters of the Southern Baltic, ranging between 8.76 and 16.17 ng·g^−1^ lw (11.68 ± 4.32 ng·g^−1^ lw), was comparable with this latter figure (Fig. 2). However, relatively recent tests performed in other parts of the Baltic recorded different concentration levels for HBCD (Bignert et al. 2011) and this almost certainly reflects the influence of the various sources by which HBCD is introduced into the environment. In Holland, for example, markedly greater concentrations (9–1110 ng·g^−1^ lw) of brominated compounds were found in fish caught in the Scheldt Estuary, probably resulting from the influence of a flame retardant plant located nearby (Janák et al. 2005).

In each fish, concentrations of brominated compounds were found to be greater in liver (HBCD 17.56 ± 3.01 ng·g^−1^ lw; TBBPA 3.27 ± 1.15 ng·g^−1^ lw) than in muscle (HBCD 11.68 ± 3.16 ng·g^−1^ lw; TBBPA 1.60 ± 0.76 ng·g^−1^ lw). This suggests prolonged exposure to HBCD and TBBPA. As with other halogenated organic compounds (Reindl et al. 2013) and mercury (Kwaśniak & Falkowska 2012), a proportion >1:1 between liver and muscle concentrations can indicate domination of detoxification overaccumulation processes within the organism.

The diet of the penguins at Gdansk Zoo consists of herring caught in the coastal zone or in the open waters of the Polish Exclusive Fishery Zone in the Southern Baltic. Each bird has a daily consumption of approximately 0.5 kg of whole herring, which contains on average 10.5 % of lipids. The average daily doses (± SD) of HBCD and TBBPA were estimated at 252.9 ± 113.7 and 26.3 ± 14.9 ng, respectively. Results presented by Falkowska et al. (2013) have already showed that daily exposure of mercury and chlorinated organic compounds by way of diet vary greatly. The performed analysis has shown the presence of brominated compounds in the tissues and organs of penguins from the zoo with the highest BFRs concentrations occurring in the brain of a relatively old female (age 8 years 7 months) (Table 1). Similar to studies of wild populations, the interactive effects of a highly complex mixture render it difficult evaluate toxicological significance of the observed brominated and chlorinated organic compounds concentration in these captive penguins.

**Table 1:** HBCD (*α*-, *β*-, *γ*-isomer) and TBBPA concentrations (ng·g^−1^ lw) in tissues and organs of African penguins from the Gdansk Zoo

Compound	Tissue and organs	Penguin age		
		8 Y 7 M	7 M	6 M
HBCD (*α*-, *β*-, *γ*-isomer)	Muscle	96.54	78.12	65.02
	Liver	131.70	90.04	70.16
	Adipose	201.13	105.78	91.09
	Brain	326.91	176.11	105.67
TBBPA	Muscle	8.92	3.78	2.77
	Liver	9.29	4.01	4.27
	Adipose	11.78	6.05	3.12
	Brain	14.78	9.91	7.12

*Y* year, *M* months

Previous results also indicated the presence of HBCD in other birds. Tests performed in 2000–2002 on the muscles of the common Baltic murre (*Uria aalge*), which feed on fish, like penguins, indicated HBCD concentrations at a level of 64.7 ng·g^−1^ lw (Lundstedt-Enkel et al. 2006). This value was comparable with those obtained from the muscles of penguins as part of the present study: 79.89 ± 15.83 ng·g^−1^ lw (Table 1). By far the highest HBCD concentrations, however, were found in the muscles of birds of prey from the British Isles: the sparrowhawk (*Accipiter nisus*) had 84–19000 ng·g^−1^ lw, whereas liver of cormorants (*Phalacrocoracidae*) contained 796–1200 ng·g^−1^ lw (Janák et al. 2008).

The performed studies have also showed the removal of BFRs from the penguins’ systems because brominated compounds were also found in the guano and unhatched eggs of these birds. HBCD concentration in guano ranged between 66.3 and 89.6 ng·g^−1^ dry weight (dw), whereas TBBPA ranged between 3.7 and 6.1 ng·g^−1^ dw. Deposition into guano confirms rapid excretion in birds and also therefore the existence of a fast-acting organism detoxification mechanism. Results obtained by other researchers were similar and showed that the removal of trace metals and POPs from bird systems is performed over short periods of time (Chen et al. 2012; Falkowska et al. 2013).

Penguins lay eggs once a year in the wild, but in the zoo there are two clutches annually. The present study measured the presence of BFRs in these eggs and found greater concentrations in yolk than in albumen. Similarly, HBCD concentrations were 10-fold greater in yolk than in albumen (Table 2). These results are consistent with previous finding concerning the lipophilicity of HBCD and BFRs (Remberger et al. 2004). Long-term studies performed by Bignert et al. (2011) indicated a constant increase in HBCD concentration in eggs of the common Baltic murre (*U. aalge*) during 1969–2009. In 2001, the content of brominated compounds ranged between 64 and 220 ng·g^−1^ lw (average 140). Lundstedt-Enkel et al. (2006) estimated mean HBCD concentration in eggs of the same species to be 138 ng·g^−1^ lw. The earliest studies, performed in 1969, recorded HBCD concentrations in the eggs of that bird that were approximately one half lower (averaging 78 ng·g_lw_^−1^); this, according to Sellström et al. (2003), suggests a gradual increase in BFR concentrations in the environment. HBCD has also been detected in eggs of others birds from different areas of Europe. The highest concentrations of brominated compounds (79–2400 ng·g^−1^lw) have been found in the eggs of the peregrine falcon (*Falco peregrinus*) in the southern part of Sweden, whereas birds of the same species in the north of Sweden had lower HBCD concentrations (34–590 ng·g^−1^ lw). The eggs of this bird in the north of Gotland, however, contained only 17 ng·g^−1^ lw HBCD (Janák et al. 2008). Mercury and other halogenated organic compounds were previously found in penguins’ eggs (Falkowska et al. 2013).

**Table 2:** Average concentration of HBCD (*α*-, *β*-, *γ*-isomer) and TBBPA in albumen, yolk, and whole egg (ng·g^−1^ lw) of African penguin from the Gdansk Zoo

Compound	Whole egg (*n* = 3)	Yolk (*n* = 3)	Albumen (*n* = 3)
HBCD (*α*-, *β*-, *γ*-isomer)	319.17 ± 95.67	245.63 ± 43.55	26.72 ± 10.67
TBBPA	11.41 ± 2.61	7.81 ± 1.98	2.72 ± 0.51

The results of the present study allowed for estimation of bioaccumulation (BAF) and biomagnification (BMF) factors for the analyzed BFRs (Table 3). For both HBCD and TBBPA, the strongest accumulation was noted for brain tissue; in addition, the adult female (8 years 7 months) showed greater factors of accumulation than the two young birds (7 and 6 months). This could suggest a greater ability to biotransform and excrete such substances by young birds than older ones. The same observation may be made for the BMF, which was also found to be greater in the adult female. However, whereas Falkowska et al. (2013) calculated a greater BMF for chlorinated pesticides in liver tissue than in muscle, the BFRs analyzed in this study were found to undergo stronger magnification in muscle than in liver (Table 3). These differing results may suggest dependence on the period of exposure.

**Table 3:** Estimated BAF and BMF values for HBCD (*α*-, *β*-, *γ*-isomer) and TBBPA in tissue and organs of African penguins from the Gdansk Zoo

Factor	Penguin age	Tissue and organs			
		Muscle	Liver	Adipose	Brain
BAF_(HBCD)_	8 Y 7 M	4.4	6.0	9.2	14.9
	7 M	3.6	4.1	4.8	8.0
	6 M	3.0	3.2	4.2	4.8
BAF_(TBBPA)_	8 Y 7 M	3.9	4.1	5.2	6.5
	7 M	1.7	1.8	2.7	4.4
	6 M	1.2	1.9	1.4	3.1
BMF_(HBCD)_	8 Y 7 M	8.3	7.5	NA	NA
	7 M	6.7	5.1	NA	NA
	6 M	5.6	4.0	NA	NA
BMF_(TBBPA)_	8 Y 7 M	9.8	5.9	NA	NA
	7 M	4.2	2.5	NA	NA
	6 M	3.0	2.7	NA	NA

*NA* tissue or organs not analyzed, *Y* year, *M* months

## Conclusion

The performed tests confirmed the presence of the main HBCD isomers as well as TBBPA in the Baltic environment. These brominated compounds were found in whole Baltic herring in their muscles and liver tissue, and a strong relationship was noted between BFRs and lipid content in all tissues.

The daily dose of HBCD administered to a penguin through its food was found to be 120.4 ng/1 kg bw, whereas a dose almost ten times lower was calculated for TBBPA (12.5 ng). This exposure through food was responsible for the presence of BFRs in the muscle and adipose and brain tissue of the penguins. The fact that the BFR concentrations were greater in liver than muscle, both for fish and penguins, highlights the importance of the liver as a detoxifying organ. However, the calculated magnification factors (BMF) indicate stronger magnification in muscle than in liver of penguins. The results showed that removal from the system takes place through guano and eggs.

HBCD and TBBPA concentrations found in fish (the penguins’ food), and in tissue and organs of the penguins themselves, were lower compared with similar data reported in literature. This may be the effect of Poland’s relatively late industrial development compared with other European countries and hence a later expansion of BFR emission sources such as insulation material plants or the textile industry. Nonetheless, the relatively high HBCD concentrations found in penguin eggs are inadequately explained by food content alone and may indicate the presence of other brominated compound sources. In Gdansk, one such source may be a plant that produces insulation materials. The question of HBCD presence in the air calls for further studies, especially regarding its presence in small aerosols (< 2.5 µm), which penetrate directly into the lungs and the bloodstream. This method of xenobiotic introduction into the system has not been investigated at all in the coastal zone of the Polish part of the Southern Baltic.
